# Unusual case of cavitary lung metastasis of esophageal cancer: A case report

**DOI:** 10.1016/j.ijscr.2021.105662

**Published:** 2021-02-18

**Authors:** Toshikatsu Tsuji, Akihiro Nishimura, Hiroki Tawara, Shinichi Kadoya, Hiroyuki Bando, Yoshio Tsunezuka

**Affiliations:** a Department of Gastroenterological Surgery, Ishikawa Prefectural Central Hospital, 2-1 Kuratsukihigashi, Kanazawa, Ishikawa, 9208530, Japan; b Department of Thoracic Surgery, Ishikawa Prefectural Central Hospital, 2-1 Kuratsukihigashi, Kanazawa, Ishikawa, 9208530, Japan

**Keywords:** CT, computed tomography, SCC, squamous cell carcinoma, AMPC, amoxicillin, AMPC/CVA, amoxicillin/clavulanate, Cavitary lung metastasis, Esophageal cancer, Total biopsy

## Abstract

**Introduction and importance:**

Cavitary lung metastases are rare. We experienced lung metastasis of esophageal cancer with a cavity, which is extremely rare.

**Case presentation:**

A 69-year-old female diagnosed with thoracic esophageal cancer underwent radical esophagectomy. Pathological diagnosis was T3N0M0-pStageII. Cavitary lesion appeared in the lower lobe of the right lung 12 months after surgery. We suspected an inflammatory change and followed up strictly. On Computed tomography (CT) image 18 months after surgery, the cavitary lesion slightly increased in size, showing wall thickening and fluid inside. Fluid collection disappeared after antibiotic treatment. The patient was followed for 4 weeks without antibiotics and fluid collection reappeared. Cavitary lesion resection for the purpose of diagnosis was planned because malignancy could not be excluded, although the readministration of antibiotics was also considered. Thoracoscopic partial resection of the right lower lobe of the lung was performed. Histopathological examination showed squamous cell carcinoma which was similar in morphology to esophageal cancer. The final diagnosis was lung metastasis of esophageal cancer.

**Clinical discussion:**

Metastatic lung cancer with a cavity is rare, accounting for approximately 4% of all cases. Moreover, Lung metastasis of esophageal cancer with a cavity is extremely rare.

**Conclusions:**

The specimen collection, including total biopsy, should be aggressively performed when diagnosing cavitary lung disease in patients with a history of neoplastic disease.

## Highlights

• Cavitary lung metastases are rare.
• This is the third report of lung metastasis of esophageal cancer with a cavity.
• Total biopsy is one of the procedures to be considered if difficulty in diagnosing in cavitary lung disease.

## 1 Introduction

In general, most cavitary lung diseases are benign. Cavitary lung metastases are extremely rare and detected in only 4% of metastatic lung nodules [1]. In practice, it is important to distinguish malignant disease from nonmalignant disease. However, it is often difficult to obtain a diagnosis.

Here, we present a case diagnosed as lung metastasis of esophageal cancer with a cavity, which is extremely rare, by total biopsy. The works has been reported in line with SCARE 2020 criteria [2].

## 2 Case presentation

**Fig. 1:**
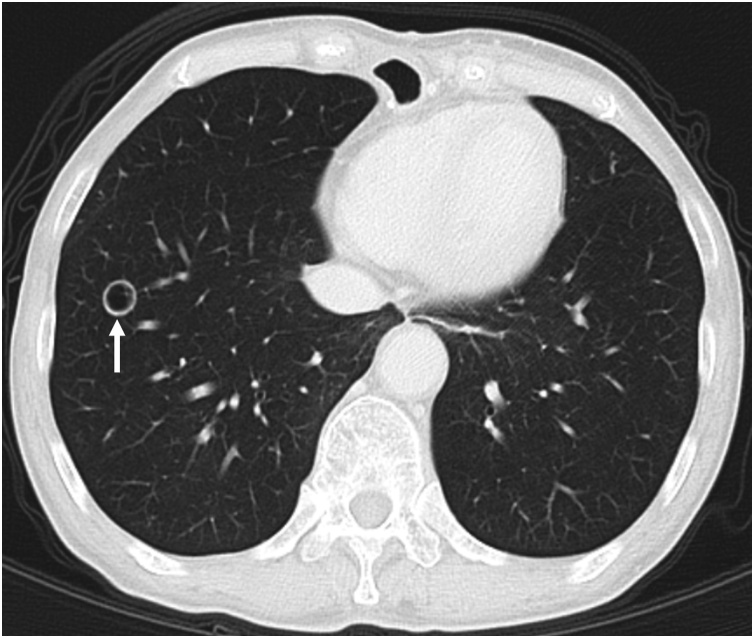
CT findings in lung window (12 months after surgery). Cavitary lesion in the lower lobe of the right lung (arrowheads).

**Fig. 2:**
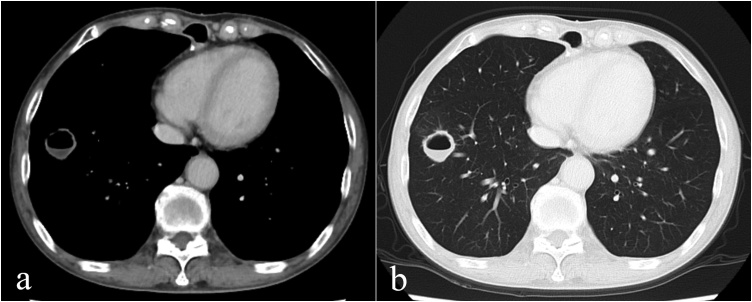
CT findings (18 months after surgery), a/b: mediastinal window/lung window. Cavitary lesion increased slightly in size, with wall thickening and fluid inside.

A 69-year-old female diagnosed with c-T3N0M0 stage II squamous cell carcinoma (SCC) in the lower thoracic esophagus underwent thoracoscopic subtotal esophagectomy with 2-field lymph node dissection after neoadjuvant chemotherapy. The pathological diagnosis was advanced esophageal cancer after induction therapy: cT grade 1a, LtAe, 25 × 25 mm, Type 2, SCC, moderately differentiated, ypT3, INFb, ly0, v3, pIM0, pPM0, pDM, ypN0, cM0, ypStage II. After that, she was followed up at our hospital. A cavitary lesion appeared in the lower lobe of the right lung 12 months after surgery (Fig. 1). This lesion was not found 6 months after surgery. We decided to follow up strictly because this lesion was suspected to be an inflammatory change rather than a tumor. Chest X-ray was planned for follow-up 3 months later, but the patient did not come to the hospital. On computed tomography (CT) images 3 months later (18 months after surgery), the cavitary lesion slightly increased in size, showing wall thickening (3.0 mm) and fluid inside (Fig. 2a,b). Infections such as tuberculosis, aspergillosis, cryptococcosis, mycosis and antineutrophil cytoplasmic antibody (ANCA)-associated pulmonary disease were strongly suspected on imaging, although a malignancy was also considered.

**Fig. 3:**
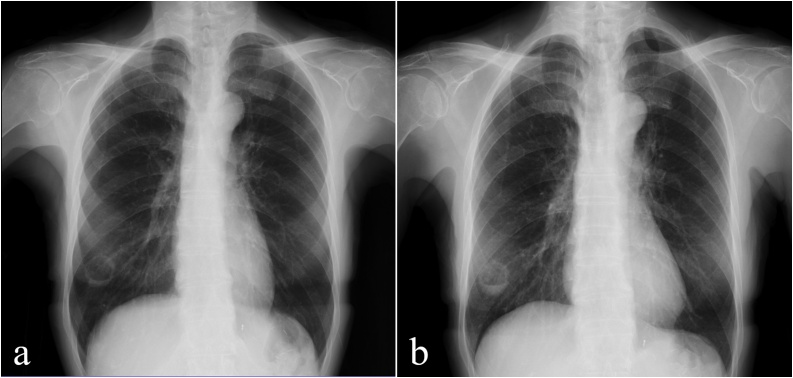
Chest X-ray findings. a: 10 days after the medication. Fluid inside disappeared. b: after 4weeks without antibiotics. The liquid in the cavity reappeared.

Regarding blood examinations, serological surrogate diagnostic markers (T-SPOT®. TB, Aspergillus antigen, Cryptococcus antigen, β-d-glucan, MPO-ANCA and PR3-ANCA) and tumor markers (SCC, CEA) were normal. Inflammatory markers (WBC and CRP) were slightly elevated. Comprehensively, bacterial infection was most suspected, and antibiotic treatment with amoxicillin (AMPC) and amoxicillin/clavulanate (AMPC/CVA) was started. Chest X-ray 10 days after medication revealed that the size of the cavitary lesion did not change and the fluid disappeared (Fig. 3a). Inflammatory markers were also normal on the blood examination. Therefore, the patient was followed without antibiotics for 4 weeks. However, the liquid in the cavity reappeared (Fig. 3b). The size of the cavitary lesion did not decrease, and malignancy could not certainly be excluded. Cavitary lesion resection for the purpose of diagnosis was planned, although the readministration of antibiotics was also considered.

Thoracoscopic partial resection of the right lower lobe of the lung was performed. Preoperatively, a point marker needle was placed near the cavitary lesion under fluoroscopy, and partial lung (S8) resection was performed using a linear stapler.

**Fig. 4:**
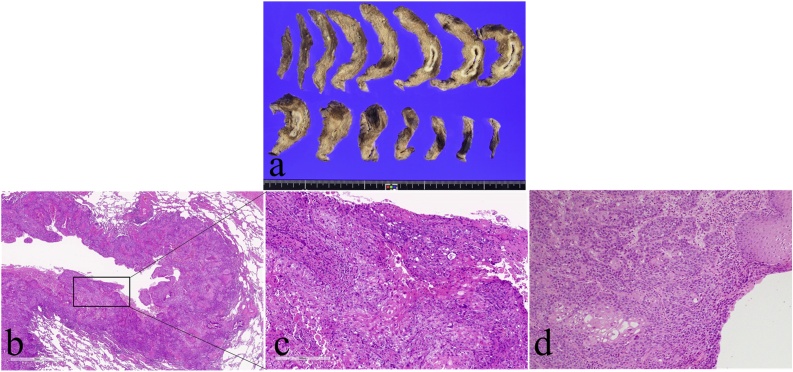
Histopathological findings. a: Resected specimens. A grayish white tumor 30 × 30 × 11 mm in size with a cavitary lesion. b: Histological findings(H.E. stain×15). c: Enlarge image. Moderately differentiated SCC(H.E. stain×100). d: Tissue specimen of esophageal cancer. Moderately differentiated SCC (H.E. stain×100).

Histopathological examination showed a grayish white tumor 30 × 30 × 11 mm in size with a cavitary lesion and moderately differentiated SCC, which was similar in morphology to esophageal cancer (Fig. 4a–d). The final diagnosis was lung metastasis of esophageal cancer.

At 6 months after the operation, there were no new recurrences.

## 3 Discussion

The lung is a common site for metastases, accounting for approximately 30–50% of all secondary locations. Metastatic lung cancer with a cavity is extremely rare, accounting for approximately 4% of all cases [1]. Approximately 70% of cavitating nodules are from SCC of the head and neck or cervix, and the rest are adenocarcinomas of the breast and colon [1,3]. To the best of our knowledge, this is the third report of lung metastasis of esophageal cancer with a cavity [4] (Table 1).

**Table 1:** Reported cases of lung metastasis of esophageal cancer with a cavity.

No.	Author	Age (y)	sex	cavity size (cm)	wall thickness (mm)
1	M. Ray Chaudhuri	64	F	1.5	4
2	M. Ray Chaudhuri	69	F	5	9
3	Our case		F	3	3

A cavity is defined as a gas-filled space seen as a lucency or low-attenuation area within a nodule, mass, or area of parenchymal consolidation [5]. It is well known that some malignant, benign, and infectious lung diseases can lead to cavitary lesions in the lungs [3]. Abscesses, mycobacterial and fungal infections and immunologic disorders are the most frequent etiologies [6]. In practice, it is important to distinguish malignant disease from nonmalignant disease. Several studies have reported a relationship between the wall thickness of the pulmonary cavity and whether it is benign or malignant [7,8]. That is, a maximum wall thickness of 4 mm or less suggests benign disease, wall thickness of 5–15 mm suggests equivalency, and wall thickness greater than 15 mm suggests malignant disease. On the other hand, primary lung cancer and pulmonary metastases are also known to rarely occur as thin‐wall cystic pulmonary lesions [7]. Moreover, pulmonary metastases occur less frequently as thin-walled cysts than primary lung cancer [9]. In our case, a cavitary lesion appeared in the lower lobe of the right lung 12 months after surgery, and the wall thickness was as thin as 1.8 mm. On the CT image after another 3 months, the cavitary lesion slightly increased in size, showing wall thickening (3.0 mm) and fluid inside.

We suspected bacterial infection because of the fluid inside and a slight increase in inflammatory markers. The fluid inside disappeared with the administration of antibiotics. Most likely, the infection was also complicated. Khalid et al [10] suggested that the duration of clinical symptoms and appearance of radiographic abnormalities can be helpful to obtain a diagnosis.

That is, an acute or subacute process (< 12 weeks) suggests common bacterial and uncommon nocardial and fungal causes of pulmonary abscesses, necrotizing pneumonias, and septic emboli, while a chronic process (≥ 12 weeks) suggests mycobacterial, fungal, viral, or parasitic infections; malignancy (primary lung cancer or metastases); or autoimmune disorders (rheumatoid arthritis and granulomatosis with polyangiitis). In the present case, the cavitary lesion appeared in 24 weeks, which corresponds to a chronic process. The definitive diagnosis of cavitary lung disease based on imaging findings is still controversial. Therefore, pathological diagnosis can be the most reliable and effective method if the diagnosis of cavitary lung disease is difficult. Fujita et al. suggested that bronchoscopy or CT-guided biopsy should be tried as much as possible when encountering cavitary lung disease [11]. We performed thoracoscopic partial resection of the right lower lobe of the lung for the purpose of total biopsy. As a result, we could obtain a definitive diagnosis without complications and dysfunction, and this operation itself could lead to a cure.

Total biopsy is one of the procedures to be considered if a case is difficult to diagnose, especially cases of suspected malignancy.

## 4 Conclusion

Cavitary lung metastases are rare and usually confused with nonmalignant diseases. Physicians should consider a potential malignancy when diagnosing cavitary lung disease in patients with a history of neoplastic disease. We should try to perform total biopsy as much as possible.

## Declaration of Competing Interest

The authors declare that they have no competing interests.

## Funding

The authors declare that they received no funding support for this report.

## Ethical approval

Ethical approval for this report has been exempted by our institution.

## Consent

Consent to publish was obtained from this patient, and the identity of this patient was protected. A copy of the written consent is available for review by the Editor-in-Chief of this journal on request.

## Author contribution

TT is the first author and prepared the manuscript under the supervision of SK and HB.

YT and TT performed the surgery. AN and HT performed perioperative therapy.

## Registration of research studies

Not applicable.

## Guarantor

Toshikatsu Tsuji, corresponding author of this article.

## Provenance and peer review

Not commissioned, externally peer-reviewed.
